# Temperature and Twist Sensor Based on the Sagnac Interferometer with Long-Period Grating in Polarization-Maintaining Fiber

**DOI:** 10.3390/s24020377

**Published:** 2024-01-08

**Authors:** Qiufang Zhang, Yiwen Zheng, Yixin Zhu, Qianhao Tang, Yongqin Yu, Lihu Wang

**Affiliations:** 1 College of Physical Science and Technology, Guangxi Normal University, Guilin 541004, China; 2021012396@stu.gxnu.edu.cn; 2 Key Laboratory of Advanced Optical Precision Manufacturing Technology of Guangdong Higher Education Institutes, Sino-German College of Intelligent Manufacturing, Shenzhen Technology University, Shenzhen 518118, China; 2110416042@stumail.sztu.edu.cn (Y.Z.); 2210412023@stumail.sztu.edu.cn (Y.Z.); 2210412014@stumail.sztu.edu.cn (Q.T.)

**Keywords:** long period fiber grating, polarization-maintaining fiber, Sagnac interferometer, temperature and twist sensor

## Abstract

We utilized a CO_2_ laser to carve long-period fiber gratings (LPFGs) on polarization-maintaining fibers (PMFs) along the fast and slow axes. Based on the spectra of LPFGs written along two different directions, we found that when LPFG was written along the fast axis, the spectrum had lower insertion loss and fewer side lobes. We investigated the temperature and twist characteristics of the embedded structure of the LPFG and Sagnac loop and ultimately obtained a temperature sensitivity of −0.295 nm/°C and a twist sensitivity of 0.87 nm/(rad/m) for the LPFG. Compared to the single LPFG, the embedded structure of the LPFG and Sagnac loop demonstrates a significant improvement in temperature and twist sensitivities. Additionally, it also possesses the capability to discern the direction of the twist. The embedded structure displays numerous advantages, including easy fabrication, low cost, good robustness, a wide range, and high sensitivity. These features make it highly suitable for applications in structural health monitoring and other related fields.

## 1. Introduction

Temperature and twist are two important evaluation parameters in the engineering application field, as traditional temperature and twist sensors, based on electromagnetic phenomena, are bulky, heavy, and usually difficult to integrate with monitored structures. Therefore, optical fiber sensors for temperature and twist have been widely studied due to their unique advantages, such as high sensitivity, strong anti-electromagnetic interference, low cost, minor size, remote sensing, and high flexibility [1,2,3]. In recent years, optical fiber sensors for temperature and twist have been widely reported based on LPFGs [4,5,6,7,8], the Mach–Zehnder interferometer (MZI) [9,10,11], and Sagnac interference (SI) [12,13,14]. For instance, Lu et al. proposed a helical sensor based on LPFG to measure temperature and twist [4]. The highest sensitivity of twist was −0.654 nm/(rad/m) in the range of −12.6~12.6 rad/m, and the sensitivity of temperature was 66.8 pm/°C from 30 to 150 °C. Zhang et al. fabricated a novel LPFG formed by tilted-arc grids (TA-LPFG) in normal simple-mode fiber [5]. The maximum sensitivity of twist is 0.514 nm/(rad/m) in the twist range of −18~18 rad/m, and the highest sensitivity of temperature is 0.0542 nm/°C from 25 to 80 °C. Among these structures, their sensitivities are relatively low and the measured ranges of twist are narrow. A sensor was fabricated by cascading two opposite helical LPFGs, which improved the sensitivity of the twist [6]. This structure achieves a high torsion sensitivity of 4.67 nm/(rad/m) in the range of −15.63~15.63 rad/m and temperature sensitivity of 0.8 nm/°C in the range of 27 to 100 °C. In addition, to obtain a double inverse helix LPFG and three transmission peaks in a certain wavelength range, they required a sophisticated control device. This suggests that the preparation of double-inverse helix LPFG is complex. Guo et al. presented a torsion sensor based on double-helix LPFG in tapered PMF [7]. They obtained a twist sensitivity of −2.28 nm/(rad/m) from −36 to 36 rad/m and a temperature of −0.106 nm/°C in the range of 25~120 °C. But the fabrication of double-helix LPFG is more complicated with a high cost. Dai et al. presented a PMF-LPFG, which was fabricated by polishing periodic complementary grooves on the principal axis of the stress of polarization-maintaining fiber [8]. The highest sensitivity of the twist was 0.81 nm/(rad/m) from −5.70 to 5.70 rad/m, and the highest sensitivity of temperature was 0.075 nm/°C in the range of 30~120 °C. Since the areas where PMF is polished are very large, the structure is greatly fragile. Therefore, it is not suitable for measurement in complex environments. Liu et al. presented an MZI by sandwiching a segment of the seven-core fiber (SCF) between two segments of multimode fibers (MMFs) [9]. This sensor has a twist sensitivity of −400 pm/(rad/m) in the range from 4.758 to 40.439 rad/m and temperature sensitivity of 123 pm/°C between 30 and 100 °C, respectively. Li et al. proposed the fiber structure by cascading with the pre-twist single-mode multimode-single mode (T-SMS) [10]. The highest sensitivities of twist and temperature are 0.32 nm/(rad/m) in the range from −11.67 to 16.27 rad/m and 71.8 pm/°C in the range from 20 to 75 °C, respectively. Ma et al. proposed a sensor based on a polished multimode single-mode multimode (MSM) [11]. It has the highest twist sensitivity of 0.196 nm/(rad/m) from −14.27 to 14.27 rad/m and temperature sensitivity of 0.072 nm/°C between 30 and 150 °C. All these structures have the following common problem: the measurement ranges of the twist are narrow, and the process of fabrication is complicated. In addition, sandwich structures are relatively fragile, which is not conducive to being used in complex engineering environments. Htein et al. presented a sensor based on a Sagnac interferometer constructed with two semicircular-hole fibers (TSHFs) [12]. They obtained the highest twist-sensitivity of 5.01 nm/° in the range of 40.36~65.86 rad/m and temperature-sensitivity of −0.16 nm/°C from 40 to 160 °C. The sensor is suitable for twist measurements in a small range. Song et al. proposed a twist sensor composed of the SI and PM-elliptical core fiber (PM-ECF) [13]. They acquired the maximum twist-sensitivity of 18.60 nm/(rad/m) between −4.36 and 4.36 rad/m and temperature-sensitivity of −0.43 nm/°C from 27.3~90 °C. They said that higher twist angles can cause measurement errors, so the twist angle is limited to −4.36~4.36 rad/m. Shao et al. proposed a torsion and temperature sensor by inserting two sections of high-birefringence (HiBi) fibers into the Sagnac loop [14]. They obtained a higher temperature sensitivity of −17.99 nm/°C and proved that the fringe visibility and torsion angle conformed to the sine relationship. This study shows that temperature sensitivity can be greatly improved by inserting the HiBi fiber into the Sagnac loop. Inspired by Ref. [14], in this paper, we inserted the LPFG written along the fast axis into the Sagnac loop, and the sensitivities of temperature and twist were significantly promoted compared with the single LPFG. The temperature and twist sensitivities were improved by about 2 and 22 times, respectively. Furthermore, through the analysis of wavelength drift direction, the sensor could distinguish the direction of twist. Due to its easy fabrication, low cost, firmness, wide range, and high sensitivity, the proposed sensor has a wide application in the inspection of structural health, such as the monitoring of oil and gas pipelines, health, and so on.

## 2. The Fabrication of PM-LPFG

**Figure 1:**
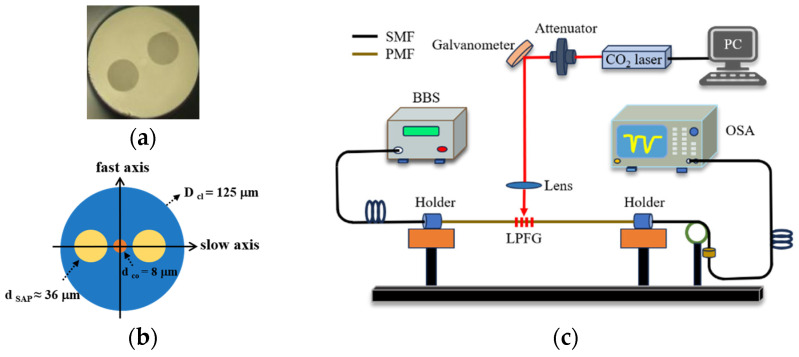
(**a**) The microscope photo of the transverse section for PMF; (**b**) Schematic diagram of the transverse section for PMF; (**c**) The schematic diagram of the experimental fabrication and measurement setup of LPFG.

The Panda PM1300-XP was obtained from Thorlabs Inc., and the transverse section microscope photo and schematic diagram are presented in Figure 1a,b. There are three different regions as follows: a germanium-doped core, the two boron-doped stress regions, and silica cladding. It has a core diameter of 8.0 μm, a cladding diameter of 125.0 μm, a stress-applying parts (SAPs) diameter of 36.0 μm, and a Numerical Aperture (NA) of 0.12. The distance between the centers of two SAPs is about 28.5 μm. Figure 1c shows the schematic diagram of the experimental fabrication of LPFG. It consists of a 10.6 μm CO_2_ laser (48-1, SYNRAD) and two fiber holders. In addition, a horizontal microscope is used to observe the writing process in real-time. The transmission spectrum of the grating is monitored using a broadband light source (BBS, Golight, SLED LIGHT SOURCE) and an optical spectrum analyzer (OSA, AQ6370D, Yokogawa) with a resolution of 0.02 nm. In step 1, a PMF with a length of 20 cm was used to determine the fast or slow axis via a special PMF coupling system (PMF-425P-OHV). In step 2, the PMF was spliced between two single-mode fibers (SMFs), and a part of the PMF coating was stripped off along the axial direction. In step 3, the bare section of the PMF was exposed to the CO_2_-laser beam. Simultaneously, we rotated the fast or slow axis of the PMF to face upwards. In step 4, one side of the PMF was clamped using a fiber holder, while the other end was fixed and affixed with a weight of 20 g to ensure the straightness of the PMF during the writing process. In step 5, we adjusted the focal plane of the laser to align with the exposed fiber using the red-light indication of the co-optical axis. In step 6, the laser beam was controlled to scan PMF using a line-by-line technique at a controllable speed. Simultaneously, a microscope was used to observe the scanning process in real-time.

**Figure 2:**
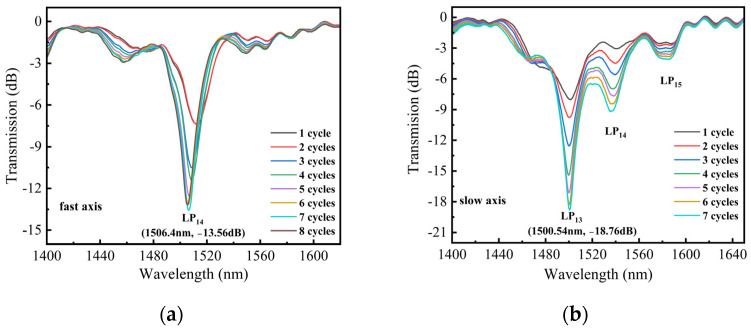
(**a**) The transmission spectrum of the LPFG irradiated along the fast axis; (**b**) The transmission spectrum of the LPFG irradiated along the slow axis.

**Figure 3:**
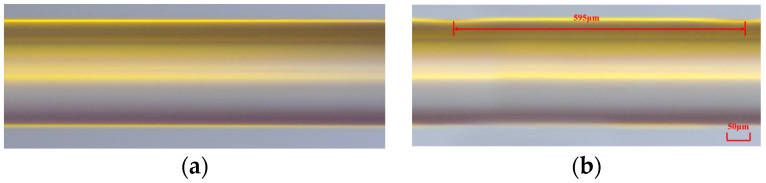
(**a**) The micrographs of PMF before irradiating; (**b**) The micrographs of PMF after irradiating.

**Figure 4:**
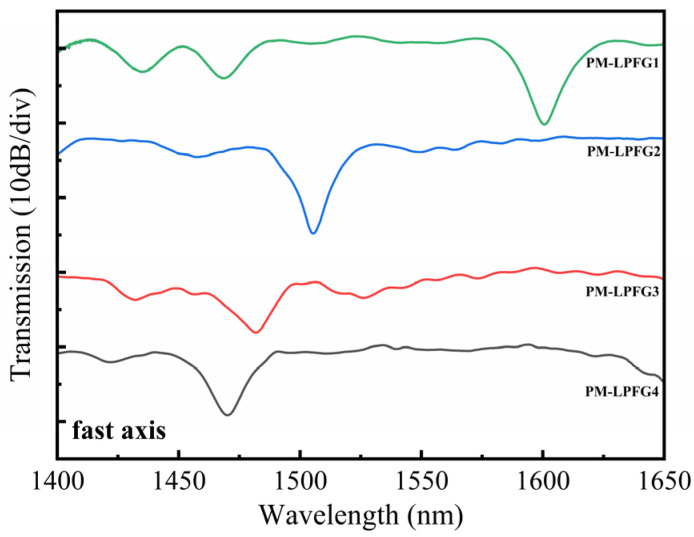
The transmission spectrum of PM-LPFGs.

In our work, the average power of the CO_2_ laser and the frequency of the laser pulses were 2.78 W and 20 kHz, respectively. Figure 2a,b display the transmission spectra of two LPFGs that were irradiated along the fast and slow axis, respectively, under various scanning cycles. The grating pitch for both LPFGs is 595 μm, and the length is 29.75 mm, which corresponds to 50 grating periods. In Figure 2a, after 1 scanning cycle, the depth of the resonance dip is 7.4 dB. With the increase in the scanning cycle, the depth of the resonance dip gradually increases. When the scanning cycle reaches 7, the depth of the resonance dip is 13.5 dB. In Figure 2b, when the scanning cycle increases, the depth of the resonance dip also increases from 8 to 18.8 dB. Furthermore, there are no new dips observed in the spectral range of 1400–1650 nm as the scanning cycles increase. After 7 scanning cycles, the depth of the resonance dip reaches its maximum value. If the number of scanning cycles is increased further, the depth gradually decreases. This phenomenon suggests that the formant mode is over-coupled. The LPFG, which is written along the fast axis, exhibits a formant depth of 13.56 dB at 1506.4 nm, where its core fundamental mode is coupled to the LP_14_ cladding mode. For the LPFG written along the slow axis, the core fundamental mode is coupled to the LP_13_ cladding mode and the resonance peak, with a formant depth of 18.76 dB, located at 1500.54 nm. Figure 3a,b display the microscopic images of PMF before and after laser inscription. After laser inscription, the surface of PMF exhibits some small grooves. The mechanism of writing LPFG on PMF (PM-LPFG) using the CO_2_ laser is mainly to release residual stress in SAPs. When PMF is irradiated along the fast axis, the laser can directly impact both the core and SAPs [15]. Under the same power, the refractive index modulation is more significant for the LPFG written along the fast axis. In the case of the same period, the resonance peak wavelength of the LPFG written along the fast axis is longer than that of the LPFG written along the slow axis. Comparing the spectra of gratings written along the fast and slow axes, we observed that, when written along the fast axis, the spectrum exhibits smaller insertion loss and fewer side lobes. Such a spectrum can avoid the interference of other dips and is beneficial for wavelength demodulation in sensing measurements. We also fabricated three PM-LPFGs with periods of 585 μm, 590 μm, and 600 μm, respectively. The total lengths of these PM-LPFGs were 29.25 mm, 29.5 mm, and 30 mm. Similarly, all three PM-LPFGs were formed through seven scanning cycles. Figure 4 shows the transmission spectra of four PM-LPFGs fabricated along the fast axis, with the curves representing PM-LPFG1 (600 μm), PM-LPFG2 (595 μm), PM-LPFG3 (590 μm) and PM-LPFG4 (585 μm) from top to bottom. As seen in the figure, the resonance peaks gradually shifted towards longer wavelengths as the period increased, which is consistent with the behavior of regular single-mode LPFGs.

## 3. The Sensing Characteristics of Single PM-LPFG

**Figure 5:**
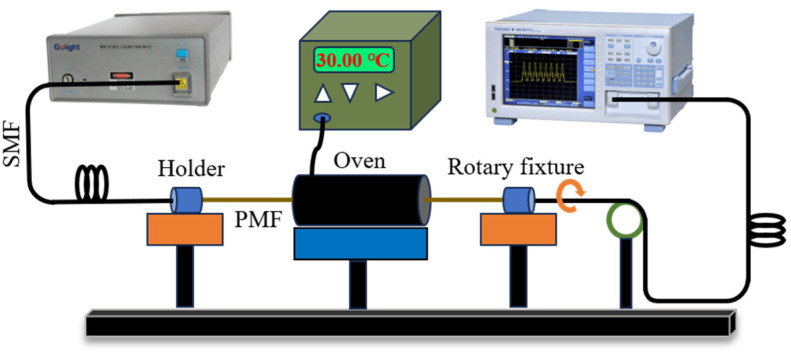
The experimental setup for PM-LPFG temperature and the twist-sensing system.

**Figure 6:**
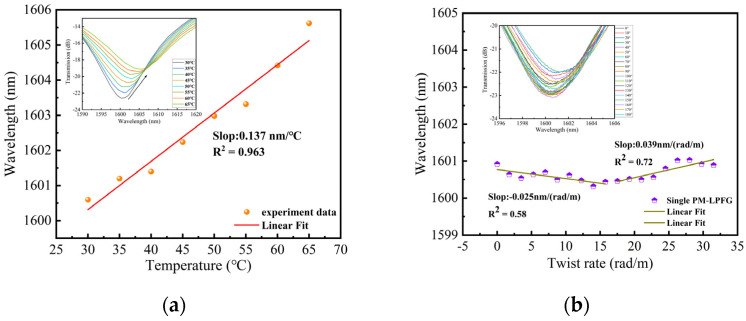
(**a**) The relationship between temperature and wavelength from 30 to 65 °C; (**b**) The relationship between twist rate and wavelength from 0 to 31.5 rad/m.

The temperature and twist characteristics of PM-LPFG1 were investigated using the experimental setup shown in Figure 5. One side of PM-LPFG1 is fixed on a fiber holder, while the other side is fixed on a rotating fixture (HFR007, Thorlabs). The distance between the two fiber holders, denoted as L, is 10 cm, and the degree of twist is denoted as θ. Therefore, the twist rate can be expressed as θ/L. The PM-LPFG1 was placed inside an oven with a temperature resolution of 0.01 °C. The temperature was controlled within the range of 30 to 65 °C, with increments of 5 °C. The sensor was kept at each temperature for 5 min, and the corresponding values from the OSA were recorded. Additionally, the twist response was examined by rotating the PM-LPFG1 from 0 to 31.5 rad/m, with increments of 1.75 rad/m. Figure 6a demonstrates that the temperature sensitivity is 0.137 nm/°C, and the inset displays the evolution spectrum of the resonance peak from 30 to 65 °C. As the temperature increases, the wavelength of the resonance dip undergoes a redshift. Figure 6b illustrates the relationship between the twist rate and wavelength, but the linearity between the twist and wavelength is not ideal. The low-temperature sensitivity of single PM-LPFG1 is attributed to the low thermal expansion coefficient of the PMF, which results in minimal length changes when the temperature varies. The reason for the low twist sensitivity is that most of the stress within the fiber is released, and the laser-induced changes in fiber size and shape are relatively small. When the twist is applied to a single PM-LPFG1, the effective refractive index difference and period variation are minimal.

## 4. The Sensing Characteristics of Embedded Structure of LPFG and Sagnac Loop

**Figure 7:**
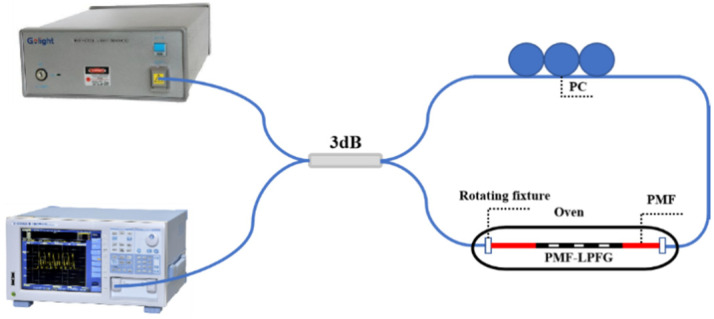
The schematic of the experimental setup for the Sagnac interferometer sensing system.

**Figure 8:**
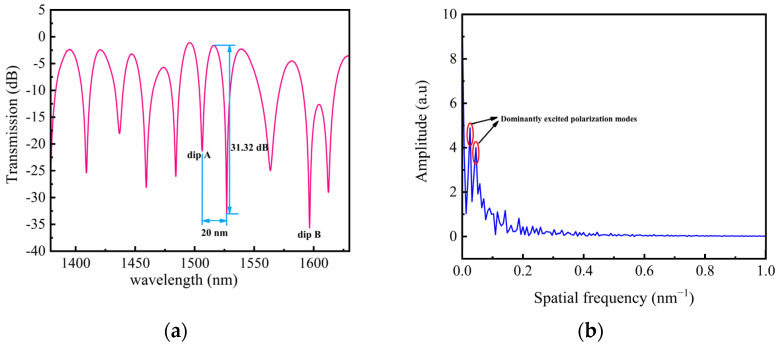
(**a**) Transmission spectra SI-based PM-LPFG1; (**b**) The FFT of the interference spectrum for SI-based PM-LPFG1.

The Sagnac loop has the advantage of high sensitivity, so we considered combining the PM-LPFG1 with the Sagnac loop to form a Sagnac interferometer (SI). Figure 7 shows the schematic of the Sagnac interferometer sensing system. In this setup, a beam of light emitted from a broadband source (BBS) was directed into a 3 dB (2 × 2) optical coupler (OC), which split the beam into two, propagating in opposite directions. When the two beams recombined at the coupler, interference occurred due to the relative phase difference introduced by the PMF. The resulting interference spectrum was recorded using an OSA. For the temperature measurement, the sensor was placed inside a temperature-controlled oven for accurate measurements. For the twist measurement, a highly precise rotating fixture was employed to twist the sensor. The all-fiber polarization controller (PC) was utilized to optimize the interference spectral pattern for enhanced accuracy. Figure 8a shows the transmission spectrum of the embedded structure consisting of PM-LPFG1 and the Sagnac loop. The interference spectrum of the Sagnac loop was modulated by PM-LPFG1, resulting in a significant decrease in spectral intensity near the resonance peak of PM-LPFG1. The interference spectrum exhibits a free spectral range (FSR) of 20 nm near 1506.3 nm. And the interference dip reached a maximum contrast of 31.32 dB at 1526.9 nm. Figure 8b illustrates the Fast Fourier Transform (FFT) of the interference spectrum for the embedded structure, revealing two prominent excited polarization modes. The spatial frequencies of these two excited modes were 0.026 nm^−1^ and 0.046 nm^−1^, with corresponding amplitudes of 4.885 and 4.006, respectively. By analyzing the sensing response of different interference valleys, we found that the interference valley with a smaller interference contrast and stronger intensity was not subjected to an external noise impact, which is convenient for twist demodulation. Therefore, we selected the interference valley at 1506.3 nm to monitor temperature and twist variations, which is denoted as dip A. Meanwhile, the resonance dip of the LPFG1 at 1596.5 nm is denoted as dip B.

## 5. Sensor Principle

For the Sagnac interference, the transmission optical intensity *I* can be expressed as follows [16]:

(1) $$I=\frac{I_{0}(1-\cos\varphi )}{2}$$

where *I*_0_ is the intensity of the input light and *φ* represents the phase difference, which is formed when two beams of transmitted light are modulated by the birefringence of PMF. And the phase difference is related to the length L of PMF and the birefringence B [17].

(2) $$\varphi =\frac{2\pi BL}{\lambda }$$

(3) $$B=\left|n_{f}-n_{s}\right|$$

where $n_{f}$ and $n_{s}$ are the effective refractive indices of PMF at the fast and slow axes, respectively. *λ* is the operating wavelength.

Interference dips may appear in the transmission spectrum when the phase difference satisfies the formula $\varphi =\left(2m+1\right)\pi$, and m is any integer. The wavelength of interference dips can be expressed as follows [18]:

(4) $$\lambda =\frac{2BL}{2m+1}$$

When the external temperature changes due to the thermal optical effect and thermal expansion coefficient of PMF, the birefringence and length change with temperature. Moreover, the axial twist loaded into the fiber also changes the birefringence and length. And temperature and twist sensitivities can be expressed by the following formula [19]:

(5) $$S_{T1}=\frac{d\lambda }{dT}=\lambda (\frac{\partial B}{B\partial T}+\frac{\partial L}{L\partial T})$$

(6) $$S_{\tau 1}=\frac{d\lambda }{d\tau }=\lambda (\frac{\partial B}{B\partial \tau }+\frac{\partial L}{L\partial \tau })$$

where $S_{T1}$ and $S_{\tau 1}$ are the temperature and twist-sensing coefficient.

For the LPFG, since the core fundamental mode of LPFG is coupled to the higher order cladding mode, the resonance dip of LPFG is formed on the existing interference spectrum. According to the phase-matching condition, the resonance dip wavelength $\lambda_{res}$ can be described as follows [20]:

(7) $$\lambda_{res}=\left(n_{eff}^{core}-n_{eff}^{clad}\right)\Lambda$$

where Λ is the grating period, and $n_{eff}^{core}$, $n_{eff}^{clad}$ are the effective refractive index of the core mode and cladding mode of LPFG, respectively.

In order to simplify the analysis of the temperature and twist response of LPFG, the variation in length is ignored. It can be considered that temperature and twist primarily alter the effective refractive index of LPFG, resulting in resonance wavelength drift. And the wavelength shift can be expressed as follows [21]:

(8) $$S_{T2}=\frac{d\lambda_{res}}{dT}=\lambda_{res}(\frac{g_{1}^{core}n_{eff}^{core}-g_{1}^{clad}n_{eff}^{clad}}{\Delta n_{eff1}})$$

(9) $$S_{\tau 2}=\frac{d\lambda_{res}}{d\tau }=\lambda_{res}(\frac{g_{2}^{core}n_{eff}^{core}-g_{2}^{clad}n_{eff}^{clad}}{\Delta n_{eff2}})$$

where $g_{1}^{core}$ and $g_{1}^{clad}$ are the thermo-optic constants for the fiber core and cladding material. $g_{2}^{core}$ and $g_{2}^{clad}$ are photo-elastic constants for the fiber core and cladding material, respectively. $\Delta n_{eff1}$ and $\Delta n_{eff2}$ represent the effective refractive index difference between the core mode and cladding mode of LPFG, which is induced by temperature and twist change. $S_{T2}$ and $S_{T2}$ are the temperature and twist sensing coefficients, respectively.

## 6. Results and Discussion

### 6.1. Temperature Performance

**Figure 9:**
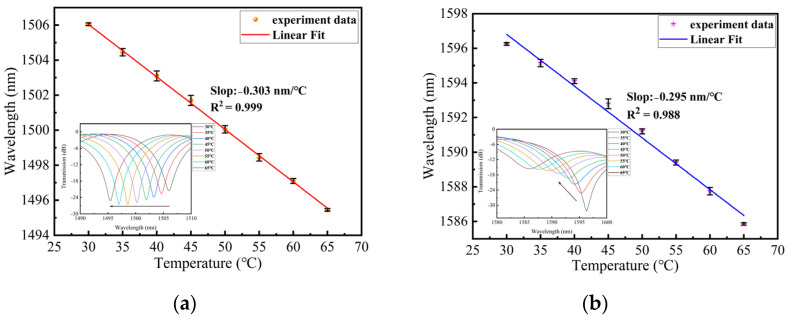
(**a**) The relationship between temperature and wavelength and error bars from 30 to 65 °C for dip A; (**b**) The relationship between temperature and wavelength and error bars from 30 to 65 °C for dip B.

The schematic of the temperature experiment, based on the embedded structure, is shown in Figure 7. The temperature of the oven was set from 30 to 65 °C with intervals of 5 °C. The embedded structure underwent testing, and the shift in wavelength showed a good linear relationship with the change in temperature, as depicted in Figure 9a,b. In these figures, the error bars represent the wavelength deviation observed during the three temperature tests. Figure 9a illustrates that the temperature sensitivity of dip A is −0.303 nm/°C within the range of 30 to 65 °C. The inset shows the evolution spectrum of dip A with temperature variation, where the wavelength shifts towards shorter wavelengths as the temperature increases. This change can be attributed to the decrease in birefringence caused by the temperature increase [22], and, according to Equation (4), the wavelength of interference dips also decreases. In Figure 9b, it can be observed that the temperature sensitivity of dip B is −0.295 nm/°C within the same temperature range. The inset depicts the wavelength of dip B blueshifts with increasing temperature. This phenomenon can be explained by the presence of two SAPs in the PMF, where the thermal expansion coefficient of the cladding is larger than that of the core. As the temperature rises, the refractive index modulation of the cladding becomes larger than that of the core [23], resulting in a decrease in the resonance wavelength of the LPFG.

### 6.2. Twist Sensor

**Figure 10:**
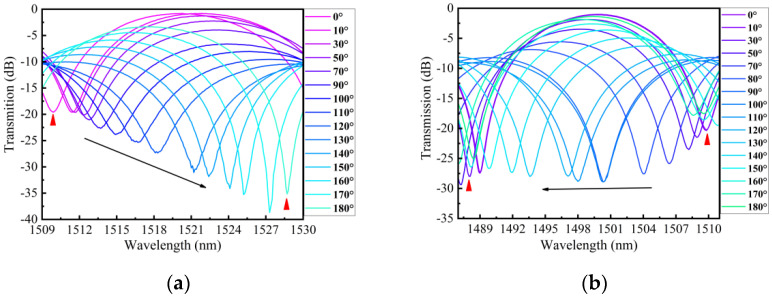
(**a**) Transmission spectrum evolution of dip A as the twist change from 0° to 180° (CW); (**b**) Transmission spectrum evolution of dip A as the twist change from 0° to 180° (CCW).

**Figure 11:**
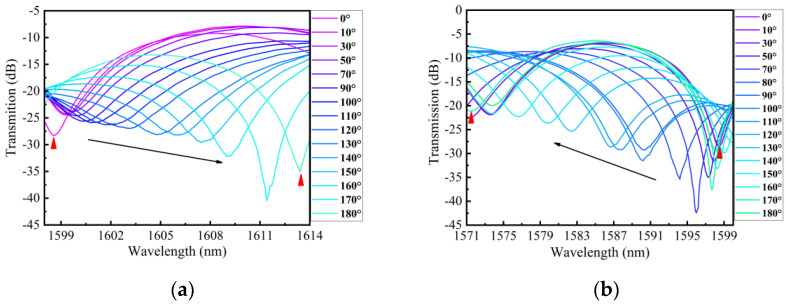
(**a**) Transmission spectrum evolution of dip B as the twist change from 0° to 180° (CW); (**b**) Transmission spectrum evolution of dip B as the twist change from 0° to 180° (CCW).

**Figure 12:**
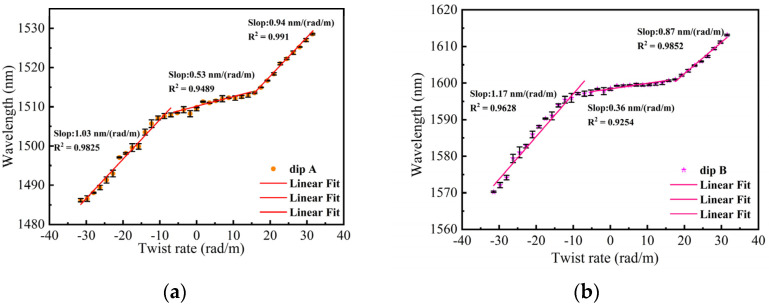
(**a**) The relationship between the twist rate and wavelength from −31.5 to 31.5 rad/m for dip A; (**b**) The relationship between the twist rate and wavelength from −31.5 to 31.5 rad/m for dip B.

The schematic of the twist experiment using an embedded structure is shown in Figure 7. One end of the structure was fixed, while the other end was twisted by a rotating fixture. Under an ambient temperature of 26 °C, the twist was applied by turning the rotating fixture from −180° to 180° with step of 10°. Figure 10a,b depict the transmission spectra evolution of 0°~180° and 0°~−180° for dip A. As the sensor was twisted clockwise (CW), the wavelength of dip A exhibited a red shift with an increasing twist angle. Conversely, when the sensor was twisted counterclockwise (CCW), the wavelength of dip A displayed a blue shift with an increasing twist angle. Figure 11a,b illustrate the transmission spectra evolution from 0° to 180° and from 0° to −180° for dip B. The wavelength variation trend of dip B was similar to dip A with the variation in the twist angle. We conducted three twist experiments using the proposed sensor within a month. In Figure 12a,b, the error bar represents the wavelength deviation of the three twist tests. From the figures, it can be observed that the repeatability of our sensor was satisfactory. As seen in Figure 12a, the wavelength shift of dip A exhibits a linear relationship with the change in twist rate for different twist ranges. Since the proposed sensor presents different sensitivities between −31.5 rad/m and 31.5 rad/m, we divided the relationship curve into three linear parts based on the variation trend of twist sensitivity. When the twist rate varies from −31.5 to −7 rad/m, the sensor has a maximum sensitivity of 1.03 nm/(rad/m). When the twist rate increases to more than −7 to 15.75 rad/m, the sensitivity is 0.53 nm/(rad/m). Finally, when the twist rate is in the range of 17.5 to 31.5 rad/m, the sensor has a sensitivity of 0.94 nm/(rad/m). Similarly, as shown in Figure 12b, the wavelength drift and twist rate of dip B also exhibit a linear relationship for different twist ranges. Through linear fitting, the twist sensitivity of dip B is calculated as 1.17 nm/(rad/m) from −31.5 to −7 rad/m, 0.36 nm/(rad/m) in the range of −5.25 to 15.75 rad/m, and 0.87 nm/(rad/m) from 17.5 to 31.5 rad/m, respectively. Furthermore, according to the linear fitting plot of the twist rate and wavelength, we can observe that when the sensor is twisted (CW), the wavelength of the interference peak shifts towards longer wavelengths, whereas when it is twisted (CCW), the wavelength of the interference peaks shifts towards shorter wavelengths. Therefore, the sensor can distinguish the direction of twist based on the direction of the wavelength shift. The twisted sensing characteristics can be attributed to the evolution of the state of polarization (SOP) caused by the twist-induced elliptical birefringence of the LPFG. This elliptical birefringence alters the SOP of the core mode and cladding modes, leading to a change in the effective refractive index between the core mode and cladding modes [24]. As shown in Equation (7), when the difference in the effective refractive index changes, the resonance wavelength also varies. The rotation direction of the SOP is dependent on the direction of the twist, allowing the proposed sensor to distinguish the direction of the twist. In addition, for the twisted PMF, assuming that the change in fiber length and the variation in instinct birefringence can be ignored, the change in the transmission wavelength can be approximately expressed as $∆\lambda =\lambda \eta b_{t}\Delta \tau$, where $b_{t}$ is a constant that describes the torsion-induced variation in the elliptical birefringence, Δτ is the change in torsion angle, and $\eta =n_{g}/B$ represents the elliptical birefringence ratio of the torsion-induced elliptical birefringence ($n_{g}$) to the sum of the fiber birefringence (B). When the PMF is twisted more, the introduced elliptical birefringence is also larger. The increase in the twist leads to an increase in η, and the twist sensitivity also increases accordingly [25].

Compared with the single PM-LPFG1 in the third section, the temperature and twist sensitivities of the embedded structure were significantly improved. Specifically, within the same temperature and twist range, the temperature sensitivity of the cascading structure increased by approximately 2 times, and the twist sensitivity increased by approximately 22 times. During the transmission process in the Sagnac loop, light beams can interact multiple times with the LPFG, which may result in coupling between the core fundamental mode and higher-order cladding modes. This mechanism can enhance the temperature and twist sensitivities of PM-LPFG1. Furthermore, the temperature and twist sensitivities of these two dips are different, indicating that this embedded structure has the potential to be used for the dual-parameter sensing of temperature and twist.

Finally, we compared the embedded structure with the sensors mentioned in the introduction, and the results are presented in the following Table 1. We primarily compared the twist range, twist sensitivity, and fabrication of various sensors. Our sensor has a larger measurement range and higher sensitivity than those in Refs. [4,5]. Compared with the double helix of LPFGs in Refs. [6,7], our sensor has the advantage of ease of fabrication and low cost. The sensors based on MZI are complicated to fabricate and have a relatively narrow measuring range and low twist sensitivity. The sensors of Refs. [12,13] have high twist sensitivity, but their measurement range is limited. The proposed sensor has the advantages of easy fabrication, low cost, good robustness, a wide range, and high sensitivity. Based on these advantages, our sensor can be widely used in complex engineering.

## 7. Conclusions

In conclusion, we wrote PM-LPFGs along the fast and slow axes using point-to-point CO_2_ laser writing technology. According to the grating spectra, we found that the spectrum of PM-LPFG, when irradiated along the fast axis, has smaller insertion loss and fewer side lobes, which is more conducive to sensing and measurement. In addition, we also explored the temperature and twist characteristics for single PM-LPFG and the embedded structure of SI and PM-LPFG. The experimental results show that the temperature sensitivity of the embedded structure is increased by about 2 times, and the twist sensitivity is increased by about 22 times. Since the temperature and twist sensitivities of the two dips are different, the embedded structure can be used for the dual parameter sensing of temperature and twist. Meanwhile, it can also identify the direction of the twist. As the embedded structure has the advantages of simple fabrication, low cost, strong structure, wide range, and high sensitivity, it exhibits great potential applications in civil engineering infrastructure, structural health monitoring, and so on.

## Figures and Tables

**Table 1:** Comparison of optical fiber twist sensor performance.

Principle	Ref	Fiber Structure	Twist Range (rad/m)	Sensitivity [nm/(rad/m)]	Fabrication
LPFG	[4]	H-LPFG	−12.6~12.6	−0.654	Hard
	[5]	TA-LPFG	−18~18	0.514	Easy
	[6]	Double inverse helix LPFG	−15.63~15.63	4.67	Hard
	[7]	Double-helix LPFG in taper PMF	−36~36	−2.28	Hard
	[8]	PMF-LPFG	−5.7~5.7	0.81	Easy
MZI	[9]	Sandwich structure in SCF	4.758~40.439	0.123	Hard
	[10]	T-SMS	−11.67~16.27	0.32	Hard
	[11]	Polished MSM	−14.27~14.27	0.196	Hard
SI	[12]	SI and TSHF	40.36~65.86	47.17	Easy
	[13]	SI and PM-ECF	−4.36~4.36	18.60	Easy
	Our work	SI and PM-LPFG	−31.5~−7 −5.25~15.75 17.5~31.5	1.17/1.03 0.53/0.36 0.94/0.87	Easy

## Data Availability

No new data were created or analyzed in this study. Data sharing is not applicable to this article.
